# A CD44-specific peptide, RP-1, exhibits capacities of assisting diagnosis and predicting prognosis of gastric cancer

**DOI:** 10.18632/oncotarget.16275

**Published:** 2017-03-16

**Authors:** Weiming Li, Huan Jia, Jichang Wang, Hao Guan, Yan Li, Dan Zhang, Yanan Tang, Thomas D. Wang, Shaoying Lu

**Affiliations:** ^1^ Department of Vascular Surgery, The First Affiliated Hospital of Xi’an Jiaotong University, Xi’an, Shaanxi Province, 710061, P.R.China; ^2^ Department of General Surgery, The First Affiliated Hospital of Xi’an Medical University, Xi’an, Shaanxi Province, 710077, P.R.China; ^3^ Department of Gastroenterology, The First Affiliated Hospital of Xi’an Jiaotong University, Xi’an, Shaanxi Province, 710061, P.R.China; ^4^ Division of Gastroenterology, Department of Internal Medicine, University of Michigan, Ann Arbor, MI 48109, USA

**Keywords:** RP-1, CD44, gastric cancer, diagnosis, prognosis

## Abstract

Early diagnosis and evaluation of prognosis are both crucial for preventing poor prognosis of patients with gastric cancer (GC), a leading cause of cancer-related deaths worldwide. Cluster of differentiation 44 (CD44), an indicator of cancer stem cells, can be specifically targeted by molecular probes and detected in tissues of GC in a large quantity. In current study we found that RP-1, a specific peptide binding to CD44 protein, exhibited the potentials of specific binding to CD44 high-expressing cancer cells both *in vitro* and *in vivo*, and the capacity of predicting prognosis of human GC in a microarray assay. Results showed that RP-1 was characterized by high affinity, sensitivity and specificity, and low toxicity, suggesting RP-1 could be an ideal bio-probe for accessory diagnosis of GC. Further immunohistochemical studies and statistical analysis of tissue microarray of human GC demonstrated similar sensitivity and specificity of RP-1 with the monoclonal anti-CD44 antibody in the diagnosis of GC, and even proved that positive RP-1 could be an independent risk factor. Therefore, this study suggests RP-1 has the potentials of binding to CD44 protein expressed on the membrane of GC cells, and demonstrates the feasibility and reliability of its further application in molecular diagnosis and prognostic prediction of GC.

## INTRODUCTION

Gastric cancer (GC) is one of the leading causes of cancer-related deaths worldwide. There are an estimated 1,000,000 new cases of GC and 723,000 cases of GC-related deaths each year [1]. The conventional white light gastroscopic biopsy and histology studies that play a critical role in GC diagnosis are facing huge challenges due to limitations of sampling error and labor intensity [2–5]. These disadvantages of traditional diagnostic methods have been hindering GC from being diagnosed at early stages and resulting in a poor prognosis, where there was no significant improvement in the last 35 years [6]. As has been demonstrated, if GC can be accurately diagnosed the first time malignancy is suspected, curative treatments can be provided to get a better prognosis [7, 8].

The combination of molecular probes and gastrointestinal endoscopy has emerged as a new method to diagnose and predict the prognosis of gastrointestinal neoplasms [9–11]. Peptide probes consisting of only a few highly specific amino acids exhibit numerous advantages, including high affinity, rapid binding kinetics, short blood-clearance time and low immunogenicity, and have technological advantages in detecting malignant lesions [12]. It has been well documented that some diagnostic markers expressed by various cancers can be specifically bound by targeted peptides. In the last decade, a number of molecular markers including cluster of differentiation 44 (CD44), have been identified in the tissue of GC, and shown clinical association with poor prognosis of GC [13–17]. However, previous reports mainly focused on the diagnostic value of peptide probes but not studied their predictive value in prognosis.

CD44, a glycoprotein of cell-surface, is closely associated with tumor metastasis and cancer stem cell (CSC) differentiation [18–20], which, regardless of its variation, has been demonstrated to play an important role in the progression of malignancies including GC [21–24]. We previously reported the RP-1, a peptide screened from a 12-mer phage peptide library, can specifically bind to CD44 protein, but didn’t focus on its prognostic and diagnostic value [25]. The purpose of the current study was to validate the specificity and affinity of RP-1 in both *in vitro* and *in vivo* experiments, and to explore the diagnostic and prognostic potentials of RP-1 for GC, therefore supporting RP-1 as an ideal molecular probe for further clinical application in both diagnosis and prognosis prediction of GC.

## RESULTS

### RP-1 demonstrated high specificity and affinity to CD44 positive cells

**Figure 1:**
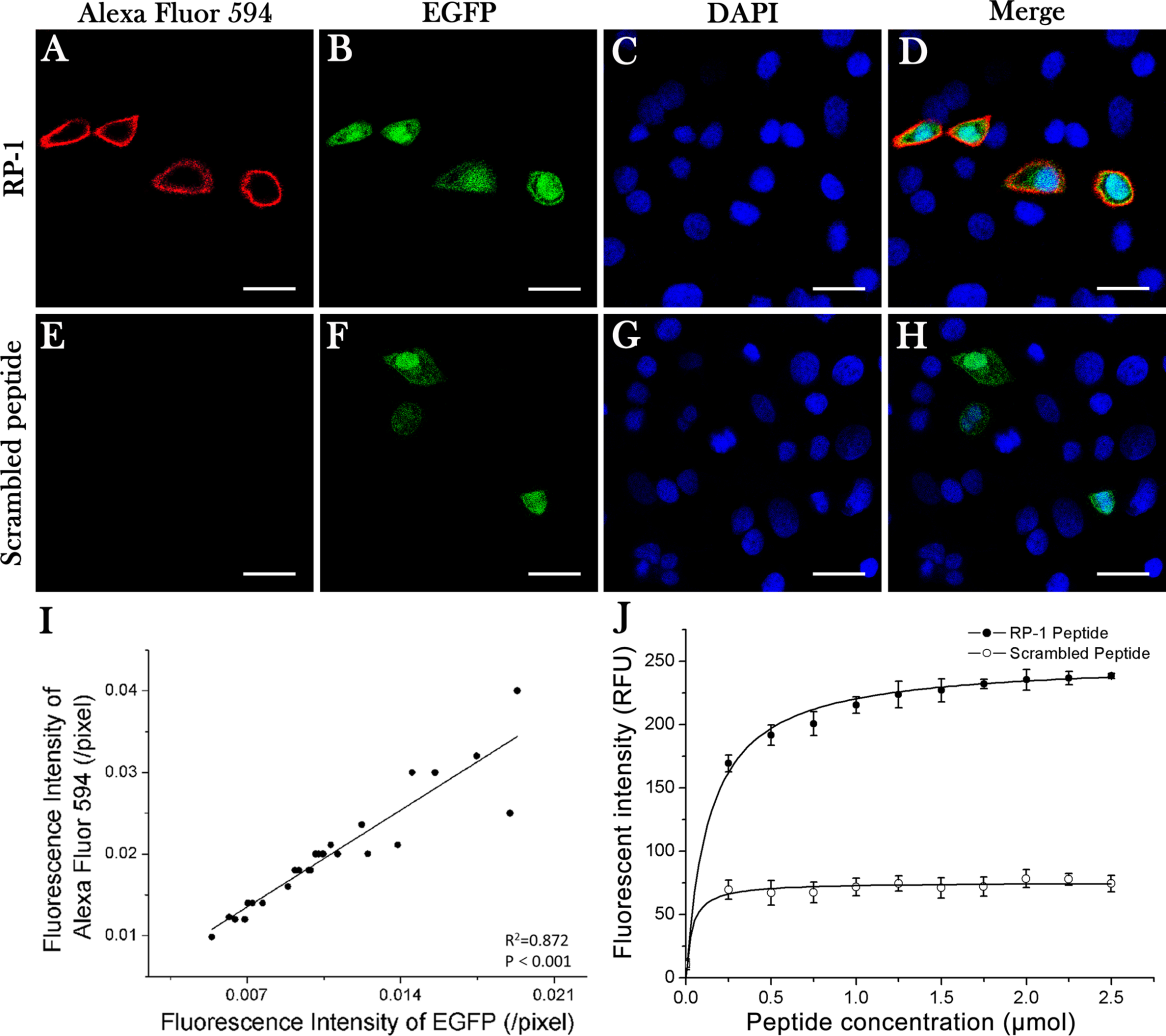
Specificity and affinity of RP-1 binding to CD44 Confocal laser microscopy images were obtained after co-cultures were incubated with Alexa Fluor 594 labeled RP-1 peptide for 20 min. (**A**) Specific binding of RP-1 peptide was found on MKN-28 cells with CD44 overexpression but not on non-transfected MKN-28 cells. (**E**) No binding of scrambled peptide was detected. (**B**, **F**) Transfected MKN-28 cells showed a green fluorescence signal of EGFP. (**C**, **G**) DAPI staining of co-cultured cells. (**D**, **H**) Colocalization (merged images) of EGFP- and Alexa Fluor 594- induced fluorescence. Scale bar, 25 μm. (**I**) A linear positive correlation between fluorescent intensities of EGFP and Alexa Fluor 594 (*R*^2^ = 0.872, *P* < 0.001). (**J**) The affinity of FITC-RP-1 to SGC-7901 cells was calculated with an equilibrium dissociation constant of K_d_ = 135 nM (*R*^2^ = 0.98), whereas no binding of FITC-WYP was detected.

Two cell lines were generated through transfection: MKN-28-con cells with no CD44 expression and MKN-28-CD44-ox cells with CD44 overexpression labeled with EGFP (Figure 1B, 1F). In these co-cultures, MKN-28 cells not expressing CD44 transgenes bound considerably lower amounts of RP-1 peptide, while those CD44-overexpressing cells had an apparent binding of RP-1 peptides to CD44 on the cell membrane (Figure 1A). No fluorescent signal was detected on cells stained with WYP (Figure 1E). Then Pearson correlation test was conducted and indicated a positive linear correlation between the binding of RP-1 and CD44 positivity (*R*^2^ = 0.872, *P* < 0.001) (Figure 1I). The results demonstrated that RP-1 could bind to GC cells through CD44 expressed on the cell membrane. A non-linear increase in fluorescent intensity of FITC-RP-1 was observed from 0 to 2.5μM, whereas the fluorescent intensity of FITC-WYP remained at a low level, which signal was considered to be non-specific (Figure 1J). The equilibrium dissociation constant (K_d_) was calculated to be 135 nM with a least squares fit, suggesting that RP-1 peptide bound to SGC-7901 cells with a high affinity.

### Low toxicity of RP-1 peptide

**Figure 2:**
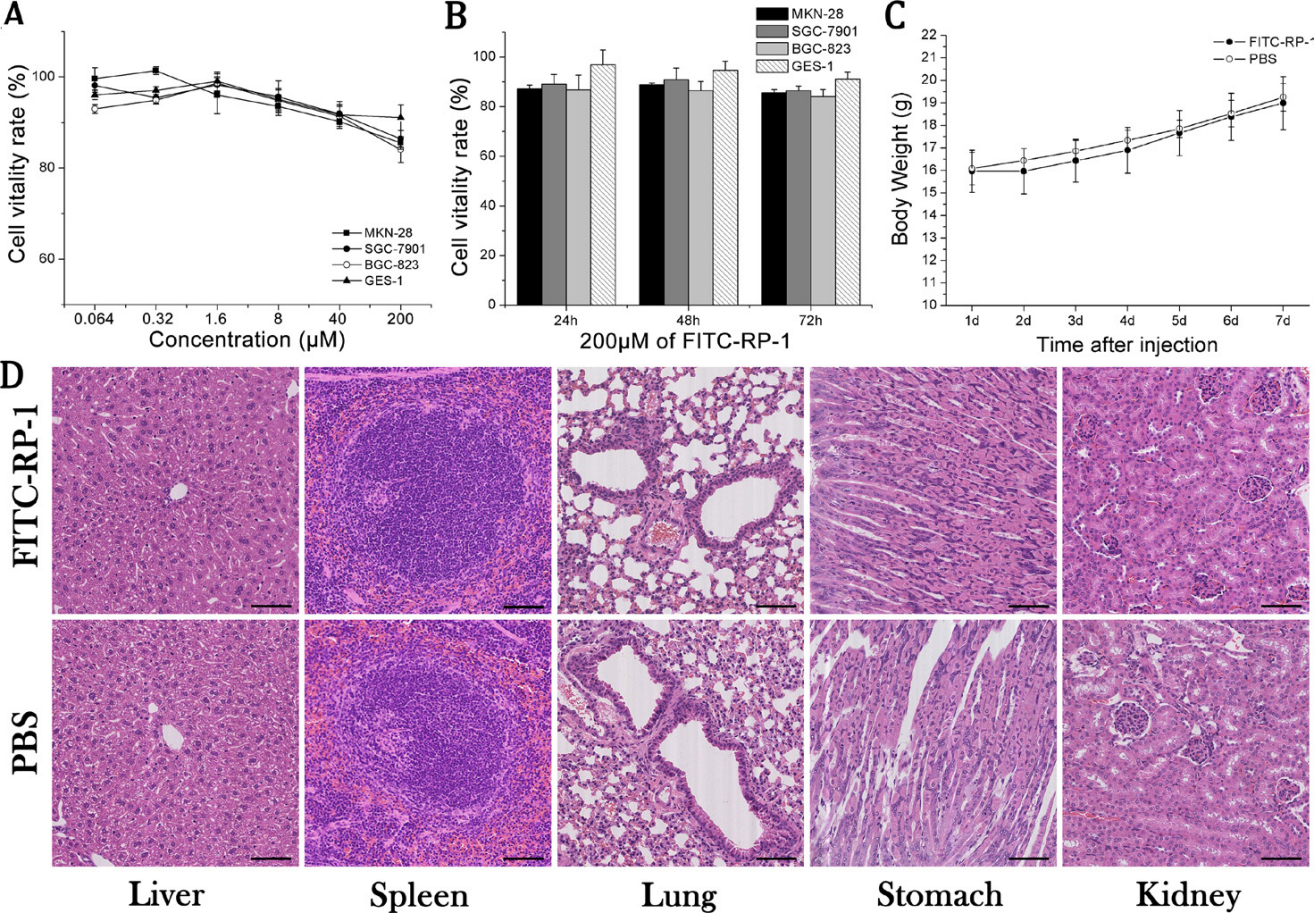
The toxicity of FITC-RP-1 *ex vivo* and *in vivo* (**A**) Cell viabilities of MKN-28, SGC-7901 and BGC-823 cells incubated with FITC-RP-1 at different concentrations for 72 h. (**B**) Cell viabilities of MKN-28, SGC-7901 and BGC-823 cells incubated with FITC-RP-1 of 200 μM at different time points. (**C**) Body weight of nude mice was measured one week after intravenous injection of FITC-RP-1. (**D**) H&E staining of normal organs harvested one week after intravenous injection of FITC-RP-1 and PBS. Scale bar, 140 μm.

Gastric cancer cells MKN-28, SGC-7901, BGC-823 and human gastric epithelial cell line GES-1 were incubated with FITC-RP-1 peptide of different concentrations for 72 h, and then cell viabilities were measured. The cell viabilities of MKN-28, SGC-7901, BGC-823 and GES-1 at the maximum tested concentration of 200 μM, were more than 85%, 86%, 84% and 91% respectively (Figure 2A). The cytotoxicity at maximum tested concentration of 200 μM was also evaluated at different times points (Figure 2B). All the results indicated that FITC-RP-1 exhibited a low cytotoxicity *ex vivo*. Then the toxicity of FITC-RP-1 peptide was tested *in vivo*. No loss in body weight was found during one week’s observation after injection (Figure 2C), and no remarkable inflammation in organs was observed from H&E staining studies in both FITC-RP-1 and control group (Figure 2D). The results of animal experiments indicated that FITC-RP-1 peptide had a low toxicity *in vivo*.

### Specific binding of RP-1 to subcutaneous xenografts *in vivo*

**Figure 3:**
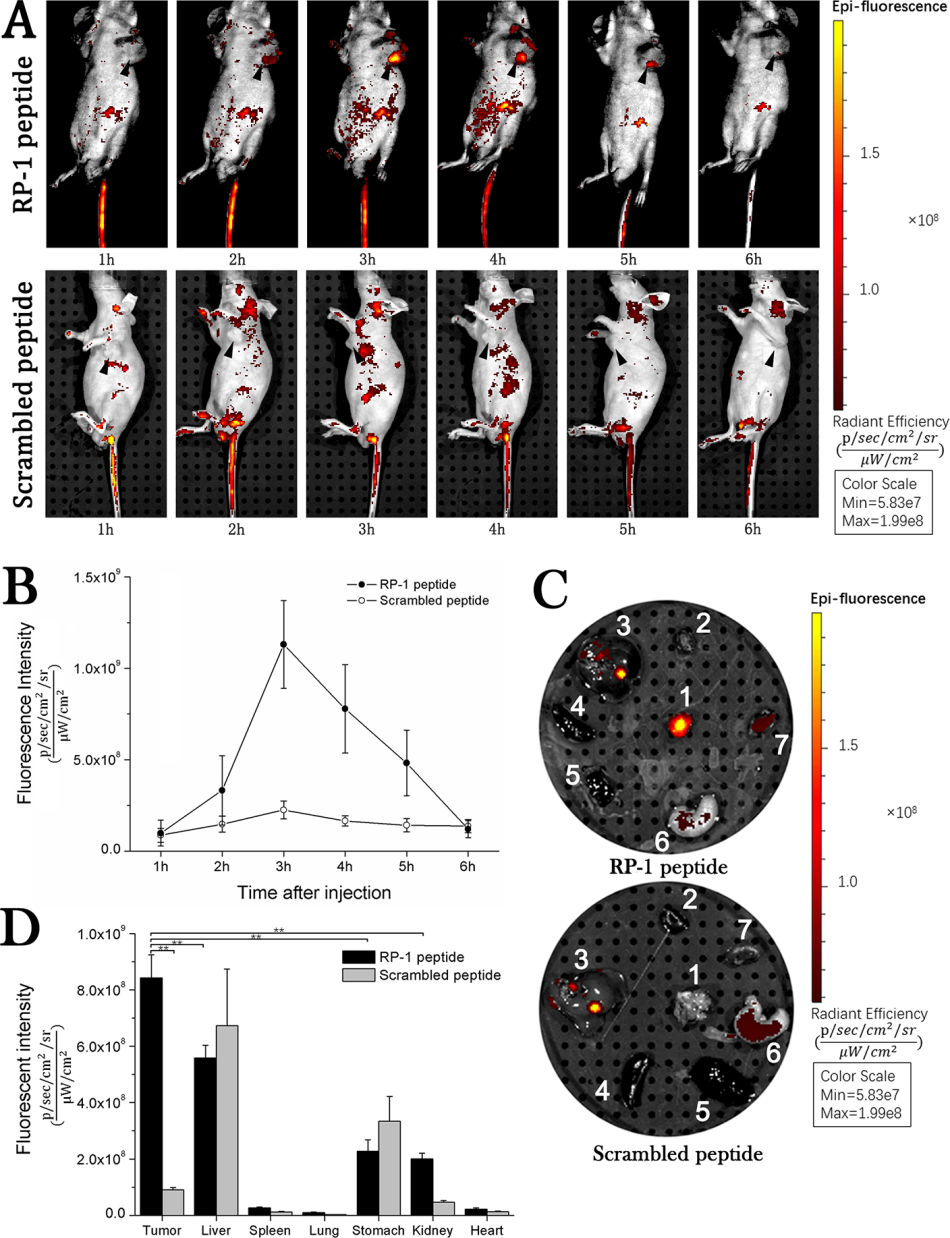
*In vivo* fluorescence imaging. RP-1 showed a high binding specificity to subcutaneous transplantation of SGC-7901 cells (**A**) Fluorescence image of nude mice subcutaneously transplanted with SGC-7901 cells by intravenous injection. (**B**) Fluorescent intensity values at ROI of tumor tissue. The accumulation of RP-1 in tumor reached its maximum at 3h, while no obvious accumulation of control peptide was observed. (**C**) Fluorescence images of excised organs (1, tumor; 2, heart; 3, liver; 4, spleen; 5, lung; 6, stomach; 7, kidney) from mice in RP-1 and control group, respectively. (**D**) Fluorescent intensity values and statistical analysis of excised organs. RP-1 had a prominent uptake in tumor tissues while only slight accumulation in normal tissues.

FITC-RP-1 and FITC-WYP were injected at a dosage of 1 μg/g body weight via tail veins in SGC-7901 subcutaneous xenograft models and fluorescence images were taken at 1 to 6 h after injection. The accumulation of FITC-RP-1 in tumor reached a peak at 3 h, while no obvious accumulation of FITC-WYP was observed (Figure 3A). Fluorescent intensity detected in gastrointestinal tract was significantly affected by the gastrointestinal contents that showed strong fluorescent signals (Figure 3A). Fluorescent intensity was quantified at the region of interest (ROI) of the tumor tissues (Figure 3B). Tumor tissues and normal organs of interest were harvested 3h after injection, and their fluorescence images showed an accumulation of RP-1 in tumor tissue and liver (Figure 3C). Then fluorescent intensity of excised tissues was quantified. In tumor, liver, stomach and kidney tissues, the fluorescent intensity values of RP-1 were 1.14 × 10^9^, 5.58 × 10^8^, 2.28 × 10^8^ and 2.00 × 10^8^ (p/sec/cm^2^/sr) /(μW/cm^2^), respectively (Figure 3D). And *t*-test on fluorescent intensity values of tumor and other organs showed a statistical significance with *P* < 0.001. Although fluorescent signal was also detected in normal organs, it was weaker than that of tumor tissue. It was speculated that the slight fluorescent signal detected in the stomach of RP-1 group might be caused by low expression of CD44 on normal gastric mucosa and non-specific binding of RP-1. Since fluorescent signal in tumor tissue was significantly higher than that in stomach, the application of FITC-RP-1 for *in vivo* GC detection would be hardly affected. Besides, fluorescence signal was almost undetectable 6h after intravenous injection, which suggested that RP-1 exhibited a property of fast elimination.

### Specificity of RP-1 binding to CD44 on tumor tissue

**Figure 4:**
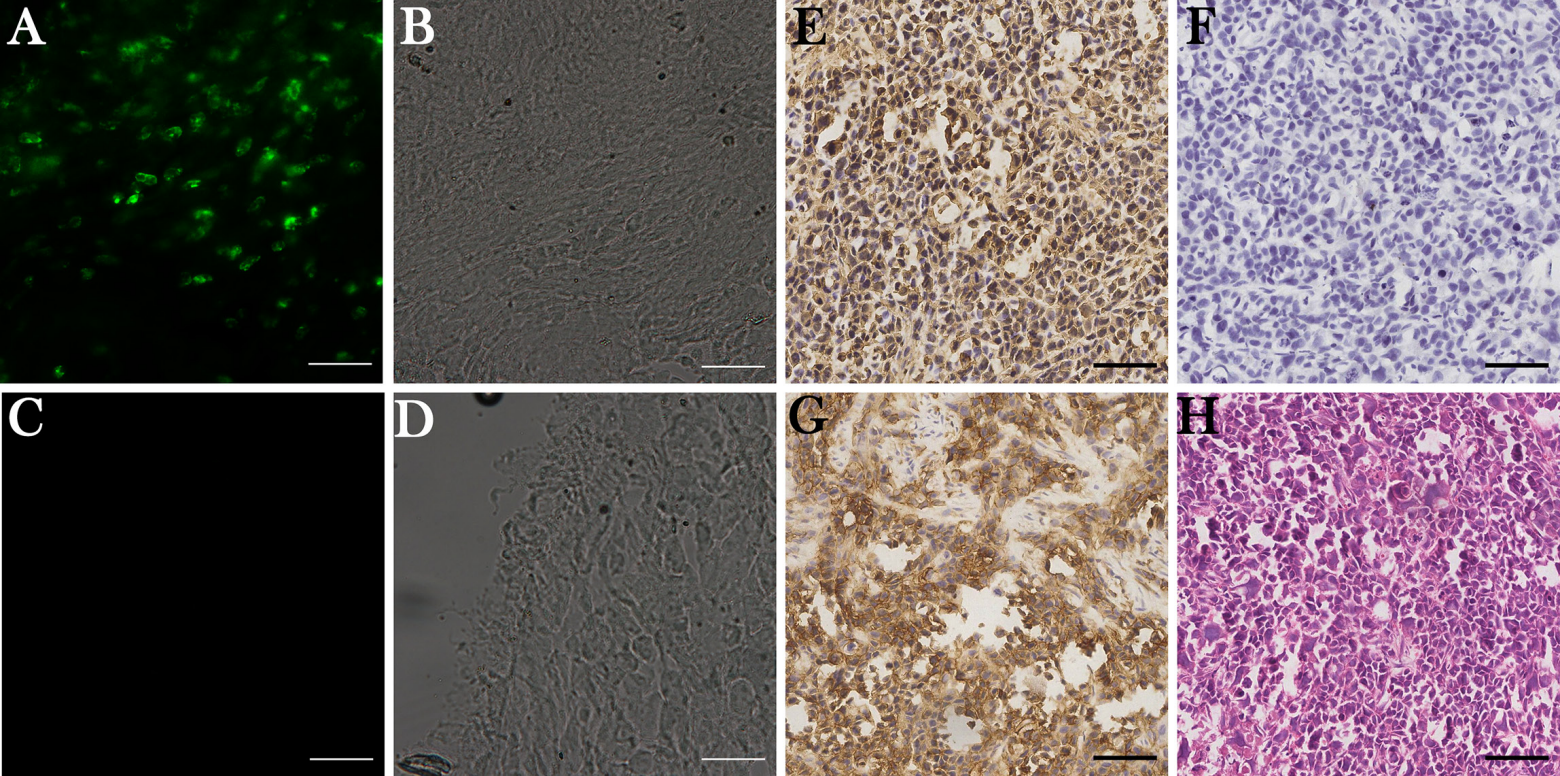
RP-1 binding to CD44 in tumor tissues (**A**–**D**) Fluorescence imaging of tumor tissue sections 3h after peptide injections. (A) Increased fluorescence signal was detected on tumor cells instead of intercellular matrix and vascular cells in RP-1 group. (C) No fluorescence signal was detected in control group. (B, D) Corresponding phase contrast images. (**E**–**G**) Immunohistochemical staining of RP-1, controlled peptide and anti-CD44 monoclonal antibody. (E) In RP-1 group, biotin labeled RP-1 had positive staining on cell membrane and in cytoplasm. (F) No obvious staining was observed for biotin labeled control peptide. (G) Positive membranous staining and cytoplasmic staining were observed in anti-CD44 antibody group. (**H**) H&E staining of tumor specimen. Scale bar, 140 μm.

Tumor tissues were harvested when fluorescence signal of tumor reached its peak and were prepared for frozen sections. Increased fluorescence of tumor cells was detected only in the frozen sections from RP-1 group, and fluorescence signals were observed both on cell membrane and in cytoplasm (Figure 4A–4D). RP-1 targeted at GC tumor cells instead of intercellular matrix or vascular cells *in vivo*. Immunohistochemistry studies showed positive staining on the cell membrane and cytoplasm in paraffin sections incubated with Biotin-RP-1 peptide or anti-CD44 antibody, indicating that RP-1 might bind to tumor cells through CD44 (Figure 4E–4H).

### Cut-off scores of RP-1 and anti-CD44 antibody

**Figure 5:**
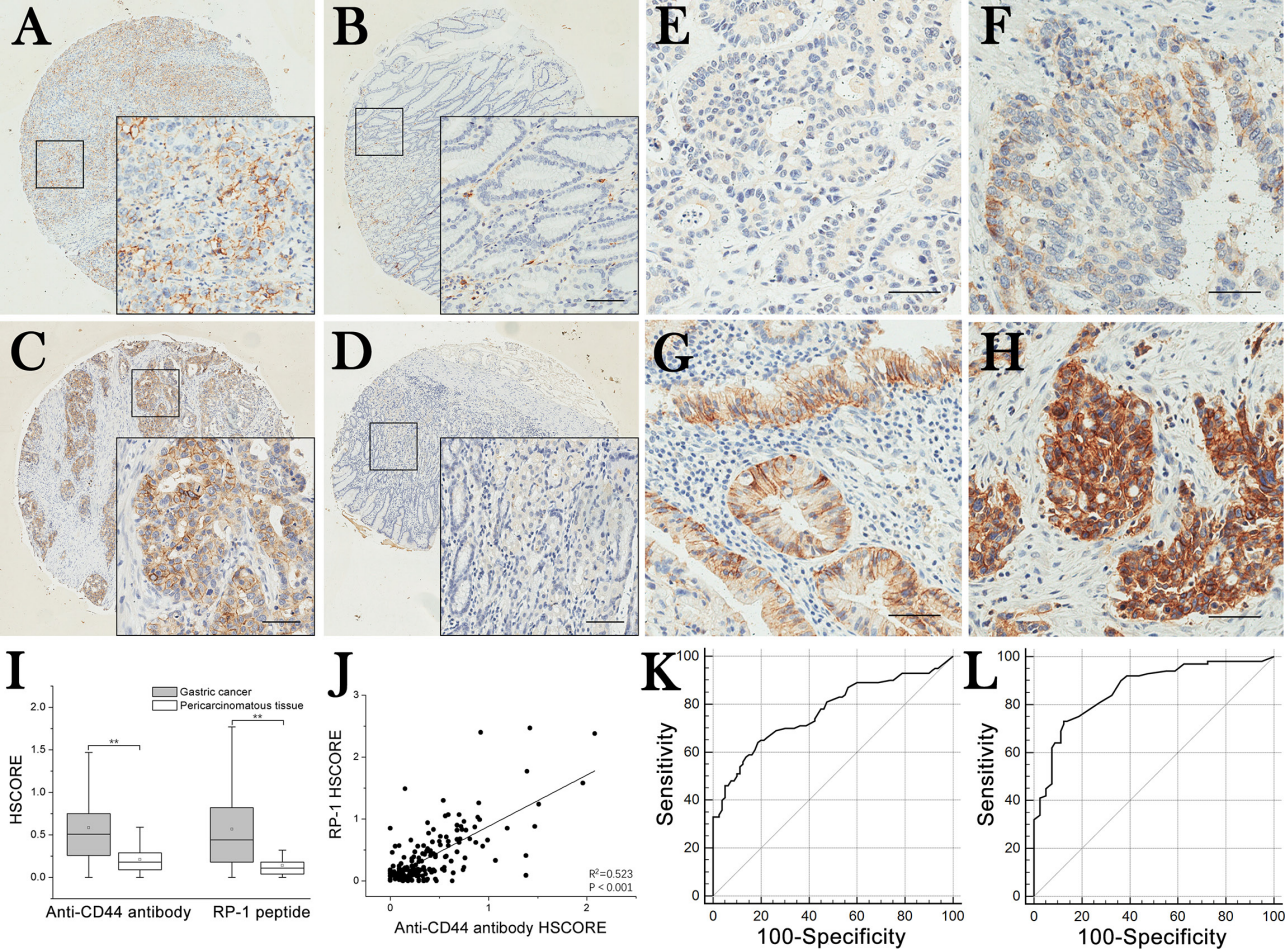
TMA immunohistochemistry staining and selection of cut-off scores (**A**) Gastric carcinoma tissues stained with anti-CD44 antibody. (**B**) Surrounding tissue stained with anti-CD44 antibody. (**C**) Gastric carcinoma tissue stained with RP-1. (**D**) Surrounding tissue stained with RP-1. (**E**–**H**) Negative (*i* = 0), weak (*i* = 1), moderate (*i* = 2) and strong (*i* = 3) positive RP-1 staining of TMA samples. Scale bar, 140 μm. (**I**) HSCOREs of gastric adenocarcimomas were significantly higher than those of surrounding tissues (*P* < 0.001). (**J**) Correlation between RP-1 and anti-CD44 antibody staining intensity. A linear positive correlation was observed between RP-1 and anti-CD44 antibody staining (*R*^2^ = 0.523, *P* < 0.001). (**K**) The cut-off score of anti-CD44 antibody was determined to be 0.33 with ROC (sensitivity, 64.0%; specificity, 81.2%). (**L**) The cut-off score of RP-1 was determined to be 0.22 with ROC (sensitivity, 73.0%; specificity, 87.5%).

In RP-1 and anti-CD44 antibody IHC staining of gastric carcinomas and surrounding tissues, immunoreactivities were observed both on cell membrane and in cytoplasm (Figure 5A–5H). Anti-CD44 antibody positive staining could be found in tumor cells and some lymphocytes (Figure 5A–5B), whereas RP-1 was positive only in tumor cells (Figure 5C–5D). Intensity of RP-1 (and antibody, figure not shown) staining of each sample was scored as 0 (Figure 5E), 1 (Figure 5F), 2 (Figure 5G) and 3 (Figure 5H). Then immunohistochemistry scores (HSCOREs) of all samples on TMA were calculated, and through *t*-test, it was demonstrated that the HSCOREs of GC samples stained with RP-1 or anti-CD44 antibody were significantly higher than that of surrounding tissue samples (*P* < 0.001) (Figure 5I). In Pearson correlation test, a linear positive correlation was observed between RP-1 and anti-CD44 antibody staining (*R*^2^ = 0.523, *P* < 0.001) (Figure 5J). The receiver operating characteristic (ROC) curves of RP-1 and anti-CD44 antibody were generated by using the SPSS software, version 21.0. The scores 0.33 and 0.20 corresponding to point (0.19, 0.64) and (0.13, 0.73), which were closest to (0.0, 1.0) and maximized in both sensitivity and specificity for diagnosis, were selected as the cut-off scores of anti-CD44 antibody and RP-1, respectively (Figure 5K, 5L). The corresponding AUCs of anti-CD44 antibody and RP-1 were 0.77 and 0.86, which suggested that both antibody and RP-1 exhibited a high diagnostic values.

### RP-1 exhibited similar sensitivity and specificity with anti-CD44 antibody

Positivity of RP-1 staining was observed in 73 gastric carcinoma tissue samples and 10 surrounding tissue samples, and positivity of anti-CD44 antibody in 64 gastric carcinoma samples and 15 surrounding tissue samples. RP-1 had a sensitivity of 73.0% and a false-positive rate of 12.5%, while anti-CD44 antibody had a sensitivity of 64.0% and a false-positive rate of 18.7% (Table 1). Result of χ^2^-test indicated that RP-1 shared similar sensitivity and specificity with anti-CD44 antibody (*P* = 0.124 and *P* = 0.403, respectively). In comparison with histological study, RP-1 and antibody had Kappa statistics of 0.592 and 0.441 respectively, suggesting that both RP-1 and anti-CD44 antibody exhibited good consistency with histological studies. Then we calculated and compared AUCs of RP-1 and anti-CD44 antibody, and found that RP-1 exhibited a diagnostic accuracy with comparable to that of anti-CD44 antibody (*z* = 2.520, *P* = 0.093). In a word, both anti-CD44 antibody and RP-1 were highly accurate in diagnosing GC and could be considered as ideal bio-probes for the molecular diagnosis of GC.

**Table 1:** The positivities of RP-1 and anti-CD44 antibody in carcinomas and pericarcinomatous tissues

		Gastric carcinomas tissues			Pericarcinomatous tissues		
		RP-1			RP-1		
		Positive	Negative	Total	Positive	Negative	Total
Antibody	Positive	55	9	64	1	14	15
	Negative	18	18	36	9	56	65
	Total	73	27	100	10	70	80

### RP-1 positivity was associated with patient survival

The positive rates of RP-1 in GC with respect to several clinicopathological variables were presented in Table 2. The results of χ^2^-test indicated that RP-1 positivity was associated with age (≤ 64.4 years vs. > 64.4 years) and patient survival status. Positive rate of RP-1 was lower in survivals than non-survivals (*P* < 0.001). However, there was no significant correlation between RP-1 positivity and other clinicopathological features including gender, WHO classification, histological differentiation, pT status, pN status, pM status and clinicopathological stage (*P* > 0.05, Table 2). Besides, RP-1 positivity could be observed in all clinicopathological stages. Furthermore, there was no statistical difference among the RP-1 positive rates in GC of different stages (*P* = 0.615, data not shown). Thus, we found that RP-1 could possibly make a role in helping to detect GC at an early stage.

**Table 2:** The relationship between RP-1 positivity and clinicopathological variables

	All cases	RP-1 staining		
		Positive (%)	Negative (%)	*P*-value
Gender				
Male	64	47 (47.0)	17 (17.0)	0.895
Female	36	26 (26.0)	10 (10.0)	
Age at surgery (years)				
> 64.4	46	34 (34.0)	12 (12.0)	0.036
≤ 64.4	54	39 (39.0)	15 (15.0)	
WHO classification				
Adenocarcinoma	66	50 (50.0)	16 (16.0)	0.182
Tubular adenocarcinoma	19	14 (14.0)	5 (5.0)	
Mucinous adenocarcinoma	8	3 (3.0)	5 (5.0)	
Signet ring cell carcinoma	4	4 (4.0)	1 (1.0)	
Undifferentiated carcinoma	3	2 (2.0)	0 (0.0)	
Histological differentiation				
Well differentiated	26	9 (9.0)	6 (6.0)	0.066
Moderately differentiated	69	53 (53.0)	21 (21.0)	
Poorly differentiated	5	11 (11.0)	0 (0.0)	
pT status				
pT1	8	5 (5.0)	3 (3.0)	0.399
pT2	9	6 (6.0)	1 (1.0)	
pT3	66	45 (45.0)	20 (20.0)	
pT4	17	17 (17.0)	3 (3.0)	
pN status				
pN0	27	21 (21.0)	6 (6.0)	0.101
pN1	15	7 (7.0)	8 (8.0)	
pN2	28	22 (22.0)	6 (6.0)	
pN3	30	23 (23.0)	7 (7.0)	
pM status				
pM0	91	65 (65.0)	26 (26.0)	0.260
pM1	9	8 (8.0)	1 (1.0)	
Clinicopathological stage				
I	10	6 (6.0)	4 (4.0)	0.526
II	32	22 (22.0)	10 (10.0)	
III	50	38 (38.0)	12 (12.0)	
IV	8	7 (7.0)	1 (1.0)	
Survival				
Died	69	58 (58.0)	11 (11.0)	< 0.001
Survived	31	15 (15.0)	16 (16.0)	

### Poor prognosis of GC was associated with positive RP-1

**Figure 6:**
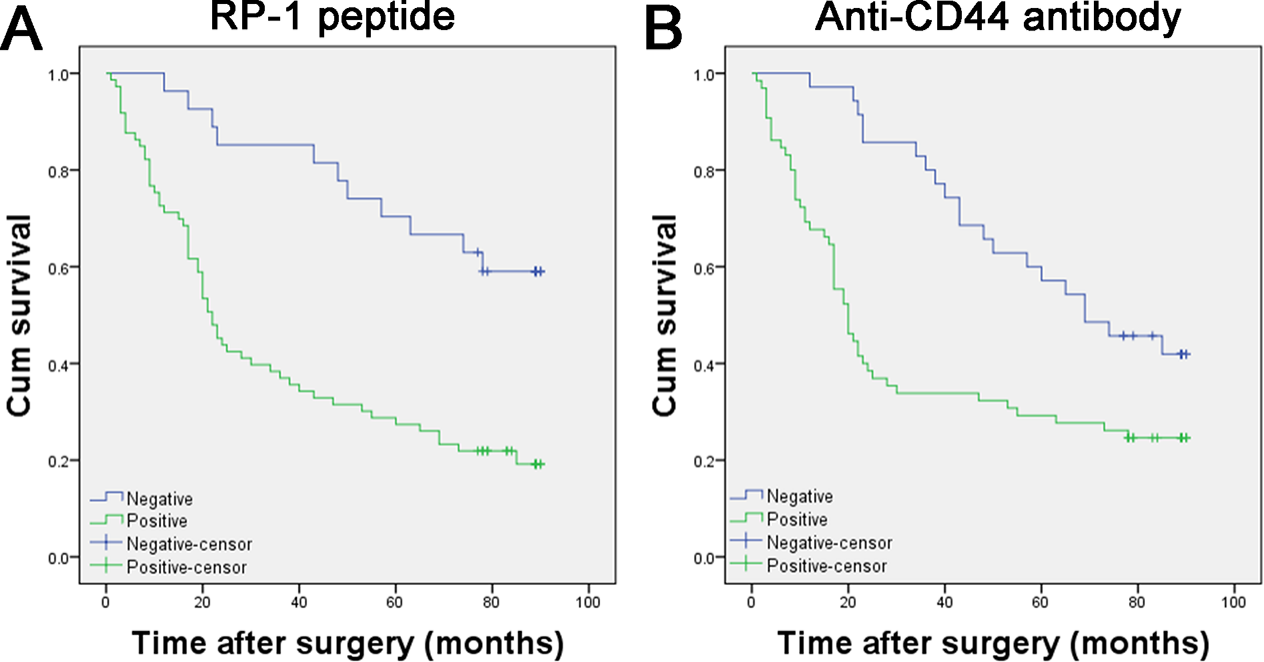
Kaplan–Meier curves for survival of gastric adenocarcinoma patients (**A**) Positive RP-1 staining was associated with poor prognosis (*P* < 0.001). (**B**) Positive anti-CD44 antibody staining was associated with poor prognosis (*P* = 0.002).

In univariate survival analysis, Kaplan-Meier survival curve was used and log-rank test was performed to calculate *P* values for determining clinicopathological variables that had significant impact on patient survival. According to Kaplan-Meier analysis, several known clinicopathological features were significantly associated with patient survival (Figure 6), such as pT status (*P* = 0.001), pN status (*P* = 0.012), pM status (*P* < 0.001), and clinicopathological stage (*P* < 0.001) (Table 3). Kaplan-Meier analysis also revealed a strong association between RP-1 positivity and antibody positivity with survival time (*P* < 0.001 and *P* = 0.002, respectively) (Table 3). The median survival time was 22.0 months in patients with positive RP-1 staining and not reached (NR) in patients with negative RP-1 staining (Table 3). Patients with positive RP-1 and antibody staining had similar median survival time (22.0 months vs. 20.0 months), but in Kaplan-Meier curve patients with negative RP-1 had a longer median survival time (of not reached) than those with negative antibody staining. Therefore, RP-1 was considered to possess the capacity to predict GC patient’s prognosis.

**Table 3:** Univariate survival analysis on clinicopathological variables and RP-1 positivity

Variable	All cases	Mean survival (months)	Median survival (months)	*P*-value
Age at surgery (years)				
> 64.4	54	41.6	23.0	0.078
≤ 64.4	46	52.2	43.0	
WHO classification				
Adenocarcinoma	66	45.1	28.0	0.154
Tubular adenocarcinoma	19	50.2	65.0	
Mucinous adenocarcinoma	8	68.4	78.0	
Signet ring cell carcinoma	4	28.0	25.0	
Undifferentiated carcinoma	3	18.0	21.0	
Histological differentiation				
Well differentiated	26	57.8	NR	0.064
Moderately differentiated	69	42.8	28.0	
Poorly differentiated	5	35.8	30.0	
pT status				
pT1	8	81.6	NR	0.001
pT2	9	52.0	63.0	
pT3	66	47.0	34.0	
pT4	17	25.2	17.0	
pN status				
pN0	27	61.5	65.0	0.012
pN1	15	57.3	69.0	
pN2	28	43.1	22.0	
pN3	30	30.8	19.0	
pM status				
pM0	91	49.8	47.0	< 0.001
pM1	9	12.9	7.0	
Clinicopathological stage				
I	10	68.3	NR	< 0.001
II	32	60.3	63.0	
III	50	38.4	21.0	
IV	8	14.3	7.0	
RP-1 staining				
Positive	73	37.3	22.0	< 0.001
Negative	27	71.3	NR	
Antibody staining				
Positive	64	36.8	20.0	0.002
Negative	36	64.6	69.0	

Notes: NR, not reached.

### RP-1 can be used as an independent predictor of GC survival

Multiple Cox proportional hazards regression was carried out to confirm the value of each variable identified in univariate survival analysis in independently predicting patient survival (Table 4). Among all the variables, pT stage (*P* = 0.027), pM stage (*P* = 0.012), antibody positivity (*P* = 0.001) and RP-1 positivity (*P* < 0.001) were found to be independent prognostic factors for the overall survival of GC patients (Table 4). Relative risk (RR) and its 95% confidence interval (CI) of RP-1 positivity was 0.284 and 0.142–0.567, which suggested that RP-1 positivity was an ideal independent predictor of survival (Table 4).

**Table 4:** Multivariate analysis on overall survival (Cox regression model)

Variable	β	Relative risk	95% Confidence interval	*P*-value
RP-1 positivity	−1.260	0.284	0.142–0.567	< 0.001
Anti-CD44 antibody positivity	−1.022	0.360	0.199–0.650	0.001
pT status				0.027
pT (1)	−3.019	0.049	0.006–0.393	0.005
pT (2)	−1.406	0.245	0.063–0.960	0.043
pT (3)	−0.522	0.593	0.330–1.067	0.081
pM status	−1.989	0.137	0.114–1.796	0.012

## DISCUSSION

Gastric cancer is one of the most common malignances with a high incidence and low 5-year survival rate due to a lack of sensitive and specific methods for in-situ detection or diagnosis. In the last decade, a number of molecular biomarkers of prognostic and diagnostic values for GC have been identified. Since the development of molecular imaging techniques in combination with endoscopy, accurate diagnosis and prediction of prognosis have gradually been more meaningful and feasible than ever. Recently, a serious of studies demonstrated the diagnostic and prognostic values of CD44 expression in human cancers including GC [26–31]. These evidences implied the possibility of predicting prognosis and making diagnosis at the early stage via molecular imaging of CD44 protein, because CD44 can be expressed in a large amount in cancer cells even at early stage of GC. As has been previously reported, RP-1 is a peptide that has both high affinity and specificity to CD44 protein [25]. In this study, we further validated the high sensitivity, specificity and affinity, and low toxicity of the targeted molecular bio-probe RP-1 by performing a series of *in vivo*, *ex vivo* and *in vitro* experiments.

Previous researches mainly focused on the biological characteristics and in-situ diagnostic values of peptide bio-probes [29, 32], but less attention has been paid to their prognostic capacities. Many factors associated with poor prognosis can be expressed by malignant cells even at an early stage. Thus, early detection of these prognostic factors may provide us with critical information regarding prognosis so that early treatment can be initiated. In this study, RP-1 exhibited capacities of both assisting diagnosis and predicting prognosis of GC, which were based on its high affinity and specificity in binding to CD44, a protein that is highly related to malignancies. Interestingly, RP-1 positive staining was observed in GC tissues from patients of all clinicopathological stages, and the positive rate had no significant correlation with clinicopathological stage. Patients with positive RP-1 staining had poorer prognosis than those with negative staining, and RP-1 positivity was proved to be an independent predictor of poor prognosis. These evidences thus indicate that RP-1 can be used in the diagnosis and prognosis prediction in GC of all stages.

With the development of imaging instruments, several optical techniques including Raman spectroscopy, optical coherence tomography (OCT), and confocal fluorescence endomicroscopy have been invented for the endoscopic detection of gastric malignant lesions [12, 33–35]. Some other studies have confirmed the prospect that the topical application of a peptide in Barrett’s esophagus *in vivo* through endoscopy, which can realize accurate early diagnosis [36]. An ideal bio-probe that can be used in endoscopic diagnosis should have such characterizations as high biocompatibility, high binding affinity, deep tissue penetration, rapid kinetics and low immunogenicity. In our study, RP-1 bio-probe with low molecular weight showed high biological affinity and low toxicity. Considering its capacities of diagnosis and predicting prognosis, RP-1 can be applied in combination with gastrointestinal endoscopy as a tool to assist diagnosis and predict prognosis at the same time.

In our study, results of tissue microarray analysis of GC patients showed that negative RP-1 staining was more associated with good prognosis compared with anti-CD44 monoclonal antibody staining, and positivity of both RP-1 and anti-CD44 antibody were comparably associated with poor prognosis. The reason may lie in the differences between the binding domains of RP-1 and antibody to CD44 protein. In a published article, the binding domain of RP-1 to CD44 protein was identified with molecular docking, which showed that RP-1 may bind to CD44 through Glu37 and Asn94 of the hyaluronan binding domain of CD44 protein [25]. However, the three-dimensional structure and special domain function of CD44 protein remain unclear. Thus, we still cannot rule out the possibility that RP-1 might bind to other domains of CD44 protein. At least, it is likely that the specific molecular domain targeted by RP-1 is closely related to GC patient’s prognosis. Further researches are needed for evaluating the diagnostic and prognostic values of the specific binding domains of CD44 protein.

There are also some limitations of our study despite the encouraging results we obtained. First, tissue auto-fluorescence can exist in *in vivo* fluorescence imaging, which may jeopardize the validity of the result. Therefore, a near-infrared fluorescein with higher efficient tissue penetration should be considered in further researches [37]. Second, although RP-1 had a relatively high sensitivity and specificity, false-positivity was still possible. To solve this issue, a bio-probe complex consisting of different bio-probes targeting at gastric carcinoma tissues can be synthesized and applied through a multi-spectral fiber endoscope with the capacity of detecting several fluorescence at different wave lengths [38, 39]. Third, the sample size of our study included only 100 cases of GC from the same geographical area and the evaluation of diagnosis and prognostic prediction may be limited by sampling error. It is necessary to verify the potential role of RP-1 using a larger sample size in further studies.

In conclusion, we have validated the specificity and affinity of RP-1 through a series of *ex vivo* and *in vivo* experiments and confirmed its value in diagnosis and prognosis prediction by tissue microarrays. The biological characteristics of RP-1 support its further clinical application as an ideal bio-probe in both accessory diagnosis and prognosis prediction of GC.

## MATERIALS AND METHODS

### Peptide synthesis

The RP-1 peptide (WHPWSYLWTQQA; ChinaPeptides Co LTD, Shanghai, China) was synthesized with the purity higher than 95%. The scrambled peptide WYP (WYPLAHWQTSWQ) consisting of the same amino acids was also synthesized as the control peptide. Peptides were labeled with fluorescein isothiocyanate (FITC), Alexa Fluor 594 or biotin at the N-terminus for further experiments.

### Cell lines

Human gastric adenocarcinoma cell lines MKN-28, SGC-7901 and BGC-823 of various differentiation grades and human gastric epithelial cells GES-1 were selected. All four cell lines were cultured in Roswell Park Memorial Institute 1640 Medium (RPMI 1640, HyClone, Utah, USA) supplemented with 10% fetal bovine serum (Gibco, California, USA) and 1% penicillin/streptomycin (HyClone, Utah, USA) and maintained in a 5% CO_2_ incubator at 37°C. All cell lines were obtained from the Cell Bank of Type Culture Collection of the Chinese Academy of Sciences, Shanghai, China.

### Establishment of CD44-expressing cell lines

Plasmids containing cDNA of CD44 protein were synthesized by GENECHEM, Shanghai, China. Using Pfu polymerase and two oligonucleotides (TCCGCTCGAGATGGACAAGTTTTGGTGGCACG and ATCGGAATTCTTACACCCCAATCTTCATGTC), the CD44 cDNA was cloned into *Xhol* and *ECOR1* sites of GV146 (GENECHEM, Shanghai, China) by recombinant PCR. Then, CD44-negative MNK-28 cells were transfected with the plasmid with Lipofectamine 2000 reagent (Invitrogen, California, USA), and then the efficiency of CD44 expression in MKN-28 cells was tested. Finally, two cell lines were generated as following: MKN-28-con with no CD44 expression and MKN-28-CD44-ox with CD44 overexpression labeled with enhanced green fluorescent protein (EGFP).

### Immunofluorescence

For the preparation of confocal laser microscopy, the transfected MNK-28 cancer cells were washed with phosphate buffer saline (PBS) three times, then fixed with 4% paraformaldehyde at ambient temperature for 30 min, and blocked with 5% BSA (w/v) in PBS at 37°C for 1 h. The blocked cells were sequentially incubated with 100 μM RP-1 or WYP labeled with Alexa Fluor 594 for 20 min, and counterstained with 4,6-diamidino-2-phenylindole (DAPI). The coverslips were mounted with glycerol–gelatin containing anti-fading buffer (Bioworld, Minnesota, USA) and examined with a confocal laser microscope (Leica, Solms, Germany). For the evaluation of the correlation of fluorescent intensities between CD44-EGFP and RP-1-Fluor 594, a semi-quantitative analysis was performed with ImageJ 1.48v software (National Institutes of Health, Maryland, USA) by randomly selecting and observing 10 fields of vision. Finally, the Pearson correlation analysis was performed by using SPSS software, version 21.0 (IBM, New York, USA).

### Measurement of RP-1’s binding affinity to GC cells

5 × 10^5^ SGC-7901 cells were inoculated into each well of a multi-well plate 24h before measurement. Both FITC- RP-1 and FITC-WYP peptide were serially diluted with RPMI 1640 culture medium containing no serum at concentrations ranged from 0 to 2.5 μM at 0.25 μM intervals, and incubated with SGC-7901 cells at ambient temperature for 15 min [40]. After washing twice with pre-cold PBS/0.2% Tween-20 solution, the unbound peptide was rinsed off, and cells were subsequently fixed in 4% paraformaldehyde at ambient temperature for 20 min. Then 200 μL PBS was added to each well for retaining moisture, and the fluorescent intensity (I) was measured with a microplate reader (Bio-Rad, Hercules, CA, USA). The equilibrium dissociation constant (K_d_) was calculated using a least squares fit of the data with Origin 8.0 software (OriginLab, Massachusetts, USA). I was calculated using the Michaelis-Menten equation *I* = *I_max_*([*X*])/(*K_d_*+[*X*]), in which *I_max_* was the maximum fluorescent intensity corresponding to peptide binding at saturation, which was calculated from the plots; *I* was the fluorescent intensity measured above; [*X*] was the concentration of total peptides and estimated to be the concentration of unbound peptide [41].

### Animal model

All animal procedures were approved by the Ethics Committee of Xi’an Jiaotong University and conducted in accordance with the Helsinki Declaration (1975). Athymic nude mice (male, 4–5 weeks old, with a body weight of 20–25 g) were obtained from the Animal Experiment Center of Xi’an Jiaotong University, Xi’an, China. The SGC-7901 tumor xenografts were generated by subcutaneous injection of 5–8 × 10^6^ SGC-7901 cells suspended in 200 μL culture medium into the left axilla of mice. The cells were allowed to grow for 2 weeks until tumor volume reached an estimate of 1 cm^3^.

### Evaluation of RP-1 peptide toxicity

Cell Counting Kit-8 (CCK-8, Beyotime, Shanghai, China) assay and animal experiments were performed to evaluate the *in vitro* and *in vivo* toxicity of RP-1 peptide, respectively. For CCK-8 assay, 5 × 10^3^ MKN-28, SGC-7901, BGC-823 and GES-1 cells in 100 μL culture medium were incubated into each well of a 96-well plate 24 h before peptide was added. All cells were incubated with FITC-RP-1 0.064 to 200 μM. After incubation for 24 h, 48 h or 72 h, 10 μL CCK-8 reagent were added into each well and further incubated for 3 h. Then, the absorbance at 450 nm was measured with a microplate reader (Bio-Rad, Hercules, CA, USA). In animal experiments, FITC-RP-1 or PBS 1 mg/kg was intravenously injected via the tail vein. The body weight of nude mice was measured every day. Then normal organs were obtained from nude mice one week after injection, fixed in a 10% formalin solution and embedded with paraffin. Hematoxylin and eosin (H&E) staining was performed for observing the histological changes. All histopathological evaluations were conducted by two independent pathologists.

### *In vivo* fluorescence imaging

*In vivo* fluorescence imaging was performed with the IVIS Spectrum Imaging System and analyzed with the IVIS Living Imaging 4.2 software (Xenogen, California, USA). Identical imaging settings (exposure time: 40s; binning: 16; lens aperture [f/stop]: 4; field of view: 13.3) were used for capturing images. Fluorescent intensity was normalized and presented as (p/sec/cm^2^/sr)/(μW/cm^2^). The mice from the experimental group (*n* = 10) and control group (*n* = 10) were intravenously injected with 1 mg/kg FITC-RP-1 and FITC-WYP, respectively. Mice anesthetized with isoflurane were subjected to optical imaging at each hour since 1h after injection. Finally, solid tumors, tissues, and organs were all harvested and rinsed with PBS for further analysis of the distribution of FITC-RP-1.

### Microscopic analysis of RP-1 peptide binding to mouse xenografts *ex vivo*

Once fluorescence signal reached its peak at the site of tumor, subcutaneous tumor tissues were harvested for further experiment. After shock-frozen with liquid nitrogen, tumor tissues were cut into 5 μm sections and fixed in 4% paraformaldehyde at ambient temperature for 30 min. The coverslips were mounted with glycerol–gelatin containing anti-fading buffer (Bioworld, Minnesota, USA). Finally, the fluorescence images of frozen sections were captured with a fluorescent microscope (Nikon-Eclipse, Tokyo, Japan)

### Immunohistochemistry

Prepared paraffin-embedded sections were deparaffinized, rehydrated, retrieved and blocked with 5% BSA (w/v) in PBS at ambient temperature for 30 min. Then, sections were incubated with 100 μM biotin labeled RP-1 peptide or human specific rabbit anti-CD44 monoclonal antibody (1:200, ZSGB-BIO, Beijing, China) overnight at 4°C. After PBS washing for three times, sections were subsequently incubated with streptavidin-HRP (1:500, Proteintech, Wuhan, China) and goat anti-rabbit secondary antibody (1:300, BOSTER, Wuhan, China). Diaminobenzidine (DAB, ZSGB-BIO, Beijing, China) was used as a chromogen. Finally, the sections were counterstained with hematoxylin and mounted with neutral balsam.

### Tissue microarrays (TMA)

Two TMA slides (Shanghai Outdo Biotech Co LTD, Shanghai, China), each containing 100 GC tissues samples (including 66 adenocarcinoma, 19 tubular adenocarcinoma, 8 mucinous adenocarcinoma, 2 undifferentiated carcinoma and 5 signet-ring cell carcinoma tissue samples) and 80 surrounding tissue samples from 100 patients, were used to evaluate the diagnostic and prognostic effects of RP-1. Samples were obtained from treatment-naive patients with written informed consent signed and dated. Age of the 100 patients with GC ranged from 32 to 81 years (mean, 64.4 years). Their clinicopathological characteristics were described in Table 2. The TNM stage of all patients with GC was assessed according to the American Joint Commission for Cancer (AJCC 7th edition) and tumors were histologically classified according to the criteria described by the World Health Organization (2000). The Medical Ethics Committee of Taizhou Hospital of Zhejiang province reviewed and approved all studies.

We performed immunohistochemical staining of TMA slides with RP-1 and human specific anti-CD44 monoclonal antibody by following the procedure described in the section *Immunohistochemistry*. The immunoreactivity was assessed with the immunohistochemistry score (HSCORE) system, which evaluated the staining intensity and percentages of positive cells stained at a specific magnitude of intensity. The HSCORE was calculated using the equation *HSCORE*=∑*Pi*(*i*) (*i* = 0, 1, 2, 3, *Pi* = 0–100%), in which *i* was the staining intensity (0, no staining; 1, weak staining; 2, moderate staining; 3, strong staining) and *Pi* was the percentage of positive cells on a scale of 0% to 100%. Positive cell count was assessed in 5 randomly selected fields of vision with a microscope (400×). All immunohistochemical evaluations were conducted by two independent pathologists who also assessed peptide toxicity as described above.

Receiver–operator curve (ROC) analysis was performed to determine the cut-off score for both RP-1 peptide and anti-CD44 monoclonal antibody. The score closest to the point (0.0, 1.0) with maximum sensitivity and specificity was identified as the cut-off score. Samples with a HSCORE of cut-off value or below were considered as negative staining, otherwise they were considered as positive staining.

### Statistical analysis

Statistical analysis was conducted with SPSS software, version 21.0 (IBM, New York, USA). Kappa values were used to determine the consistency of histological and immunohistochemical results between RP-1 peptide and anti-CD44 antibody. Independent sample *t*-test was performed to decide existence of statistical difference in fluorescent intensity values between tumor and other normal organs. The ROC analysis was carried out to decide the cut-off value for RP-1 and anti-CD44 antibody. The area under the curve (AUC) was calculated to compare the diagnostic accuracy of RP-1 and anti-CD44 antibody. χ^2^-test was used to assess the association between each clinicopathological variable and RP-1 staining intensity, while log-rank test was used for assessing the association between survival and each clinicopathological variable. Multiple Cox proportional hazards regression was performed to verify if RP-1 positivity can be used as an independent risk factor for prognosis of GC. All statistical tests were two-tailed tests and *P* value < 0.05 was considered to be of statistical significance.
